# A cross-sectional study on the age-related cortical and trabecular bone changes at the femoral head in elderly female hip fracture patients

**DOI:** 10.1038/s41598-018-36299-y

**Published:** 2019-01-22

**Authors:** Tristan Whitmarsh, Yoshito Otake, Keisuke Uemura, Masaki Takao, Nobuhiko Sugano, Yoshinobu Sato

**Affiliations:** 1 Imaging-based Computational Biomedicine Laboratory, Nara Institute of Science and Technology, Ikoma, Japan; 2 Department of Orthopaedic Medical Engineering, Osaka University Graduate School of Medicine, Suita, Japan

## Abstract

Bone is in a continuous state of remodeling whereby old bone is absorbed and new bone is formed in its place. During this process, new formations reinforce the bone in the direction of the dominant stress trajectories through a functional adaptation. In normal aging, the balance between bone resorption and formation can be shifted. How this affects the functional adaptation remains to be investigated. Furthermore, how or whether the bone continues to change beyond the age of 85 is not yet studied in detail. In this study we examined the age-related changes in the cortical and trabecular bone in old age, and assessed whether we can find evidence of the presence of functional adaptation. We measured cortical and trabecular parameters from micro-computed tomography scans of the femoral head extracted from hip fracture patients between the age of 70 and 93 years. A significant decrease in global trabecular bone mineral density (38.1%) and cortical thickness (13.0%) was seen from the 9th to the 10th decade of life. The degree of anisotropy was maintained globally as well as locally in both high and low stress regions. The local trabecular bone mineral density decreased in both high stress and low stress regions between the 9th and 10th decade of life with similar trends. This suggests that the role of functional adaptation in maintaining the bone structural integrity in old age may be limited. This study highlights the need for a controlled clinical trial examining the cause of the continued bone degradation throughout old age.

## Introduction

Normal aging is associated with a shift in balance between bone resorption and formation. This causes a net decrease in bone mass and a deterioration of bone micro-structure, which in turn makes the bones more susceptible to fractures. In post menopausal women this is largely caused by a change in the hormone levels^1^, but there may be various other causes^2^.

The law of bone remodelling, commonly referred to as Wolff’s law but more accurately described by Roux^3^, asserts that the internal trabecular bone adapts to external loading conditions to maximize its resistance to the principal stress trajectories. This functional adaptation of trabecular bone is a well established phenomenon, although the underlying mechanisms are not yet fully understood^4^.

That bone mineral density (BMD) decreases in old age is already a well known fact^5,6^. A decrease in BMD in old age, however, can not fully explain the increased risk of hip and vertebral fractures^7^. It is now becoming increasingly apparent that the change in trabecular structure that accompanies a decrease in bone mass, is more important than the amount of bone itself^8^.

With evidence emerging that hip fractures may be caused by localized bone deficiencies^9^, understanding what leads to these focal bone degradations are of paramount importance. It has been hypothesized that, while trabecular elements transverse to the primary load direction may decrease during aging, trabeculae oriented along the principal compressive axis are largely maintained due to functional adaptation. This might make a bone more susceptible to fractures at loading conditions deviating from the main direction, as experienced for instance with a fall on the hip. Indeed in the study by Ciarelli *et al*.^10^ it was shown that, compared to controls, fracture patients had fewer trabecular structures transverse to the primary load axis. This reduces the cross-bracing and thus the resistance to transverse loads. In support of this, Gao *et al*.^11^ showed that the mechanical properties in the longitudinal direction deteriorated more quickly than those in the transverse direction.

The degree of anisotropy (DA) is a common measure to indicate how oriented the bone structure is and might indicate the presence of functional adaptation. This measure has consistently been shown to increase in old age^12–14^, but not statistically significantly. How load dependent remodeling is affected by the change in balance between bone resorption and bone formation in old age, and whether this leads to different rates of bone loss between loaded and unloaded bone remains to be investigated.

In this study we investigated the bone changes and the possible influence of functional adaptation in old age using micro-CT scans of the femoral head. To study the effects of functional adaptation, the femoral head is particularly suitable due to the clearly distinguishable loaded and unloaded regions within it. The age-related changes of the trabecular BMD (Tb.BMD) and the DA was therefore analyzed globally, as well as locally at key regions within the femoral head. Bone changes due to a functional adaptation may also be expressed in the cortex. Therefore, we also examined the cortical thickness (Ct.Th) and density changes (Ct.BMD) and assessed the possible changes in uniformity which might occur due to functional adaptation.

## Materials and Methods

### Data

Femoral head specimen were obtained from patients who underwent a bipolar hemi-arthroplasty following a femoral neck fracture or an unstable intertrochanteric fracture. During this procedure the femoral heads were removed without the use of a corkscrew to avoid damaging the femoral head. They were subsequently stored in 10% formaldehyde. Each femoral head was removed from formaldehyde just before taking the micro-CT scan. Micro-CT scans were acquired of the specimen in air using a Cosmo Scan FX device (Rigaku Corporation, Tokyo, Japan) using the following settings: 90 kV; 160 μA; 73 mm field of view; 0.1476 mm slice thickness and a scan time of 8 minutes^15^. The resulting volumes consist of 0.1476 mm cubic voxels. Only the scans of female patients were used in this study. Scans of patients below the age of 70 were rejected as well as scans with insufficient intact femoral head bone tissue. No other rejection criteria were applied resulting in a dataset of 37 scans. The baseline characteristics of all subjects included in this study are presented in Table 1. This research was approved by the institutional review board at the Osaka University Graduate School of Medicine. Informed consent was obtained from all individual participants included in the study and all procedures were conducted in accordance with the Declaration of Helsinki.

### Trabecular Bone Mineral Density

For each micro-CT scan a calibration phantom (Rigaku Corporation, Tokyo, Japan) with known mineral densities was included in the field of view. A user interface allowed us to delineate regions with known densities and measure the average CT values. A linear regression then provided us with the equation to convert the CT value to a corresponding bone mineral density expressed in *mg*/*cm*^3^. The average CT values were computed within the defined regions and were in this way converted to the trabecular bone mineral density (Tb.BMD).

### Degree of Anisotropy

Otsu’s method^16^, a clustering-based image thresholding, was used to identify voxels as belonging to either bone or marrow. By sampling the voxels in the volume by a set of parallel lines, the number of bone intersections in this direction was counted. By dividing the total length of the sample lines by the number of intersections we then derived the mean intercept length (MIL)^17^. By doing this in many random directions we created a rose diagram of the MIL values, onto which an ellipsoid was fitted. The DA was then computed as 1 minus the ratio between the shortest and the longest principal axis length. This produced a value between 0 and 1 whereby a value of 0 means the bone is isotropic and a value of 1 indicates anisotropic trabecular bone.

### Local Evaluation

**Figure 1:**
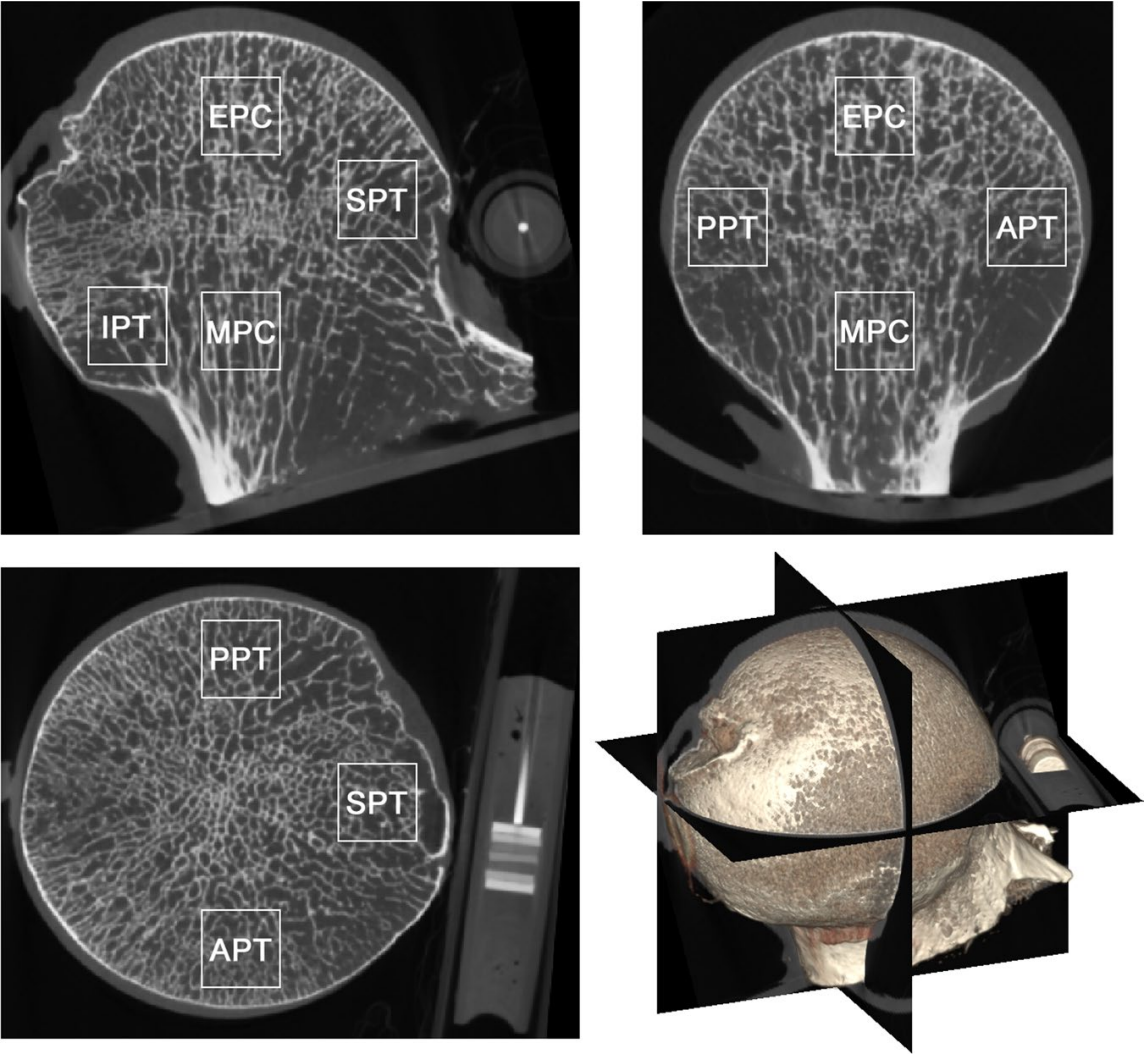
Representative femoral head micro-CT scan with the highlighted regions used in the local analysis named the metaphysial principal compressive (MPC), epiphysial principal compressive (EPC), superior principal tensile (SPT), inferior principal tensile (IPT), anterior principal tensile (APT) and posterior principal tensile (PPT) region. The micro-CT scan was resampled to align the principal compressive structure with the z-axis and the fovea capitis towards the x-axis. The calibration phantom can be seen as composed of seven discs with various intensities.

To consistently evaluate the localized regions across all subjects, the orientation of the femoral head was standardized by resampling the CT images using a method proposed by Chiba *et al*.^18^. Briefly, the principal compressive structure was aligned with the z-axis and the direction of the fovea capitis femoris was defined as the x-axis (Fig. 1). Anatomical locations within the femoral head were defined in a similar manner as Crane *et al*.^19^. At these locations a cubic region of 54 voxels corresponding to 8 mm was defined, within which the DA and Tb.BMD was measured. The regions selected for this study are listed below and illustrated in Fig. 1:

- **MPC**: the metaphysial principal compressive region in the metaphysis, immediately inferior to the epiphyseal plate.
- **EPC**: the epiphysial principal compressive region in the proximal epiphysis, superior to the epiphyseal plate.
- **SPT**: the superior (lateral) principal tensile region.
- **IPT**: the inferior (medial) principal tensile region.
- **APT**: the anterior principal tensile region.
- **PPT**: the posterior principal tensile region.

Subjects were classified as belonging to one of three age groups. From 70 till 79 years, 80 till 89 years, and between the age of 90 and 99. These were denoted the 8th, 9th and 10th decade groups respectively. As some of the femoral heads were fragmented, not all regions were available in all specimen. Also scan artefacts, air pockets or damage from the tools used in the surgical procedure caused some regions not to be included in the analysis. Table 2 reports the number of subjects in each category.

**Table 1:** Baseline characteristics (mean ± SD).

Age group	Weight (kg)	Height (cm)	YAM (%)
70–79 (n = 6)	52.4 ± 6.7	154.7 ± 4.5	70.7 ± 8.7
80–89 (n = 22)	47.1 ± 8.5	146.6 ± 7.0*	65 ± 17.0^†^
90–99 (n = 9)	44.0 ± 5.7	147.6 ± 6.5	57 ± 11.5

YAM: percentage of young adult mean measured by Digital Image Processing (DIP). *Significantly different with respect to previous age group (*p* < 0.05) assessed by two-sample T-test. ^†^Measurements were taken from 17 patients since data was not available of 5 patients.

**Table 2:** The number of subjects for each measurement location and age group.

Region	70–79	80–89	90–99
MPC (metaphysial principal compressive)	5	21	8
EPC (epiphysial principal compressive)	6	22	9
SPT (superior principal tensile)	4	16	9
IPT (inferior principal tensile)	3	20	9
APT (anterior principal tensile)	5	20	9
PPT (posterior principal tensile)	6	20	8
Global	3	12	7

### Global Evaluation

**Figure 2:**
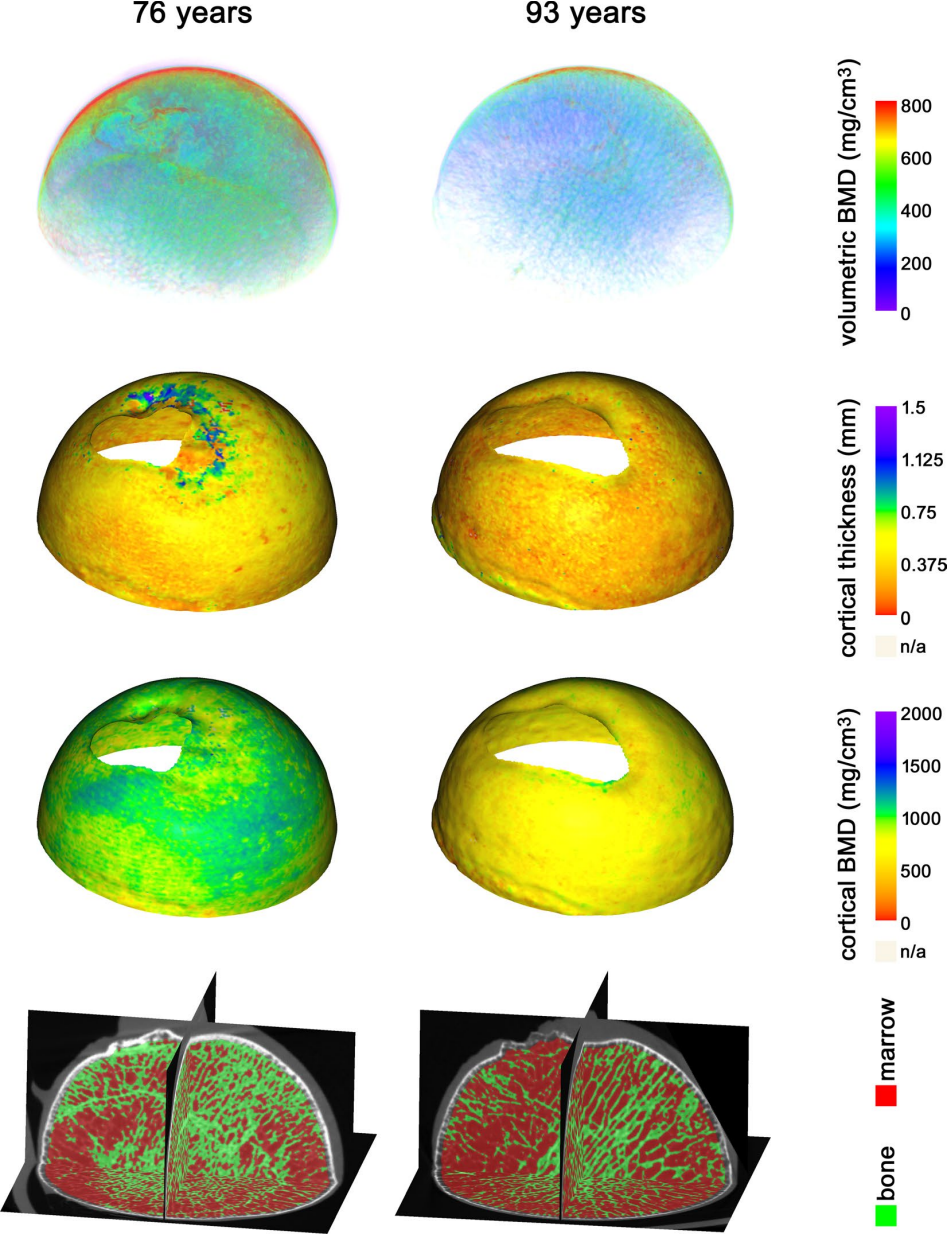
Illustrations of the micro-CT measurements from a subject of 76 yrs (Left; trabecular bone mineral density (Tb.BMD) = 221 *mg*/*cm*^3^, cortical thickness (Ct.Th) = 0.42 *mm*, cortical bone mineral density (Ct.BMD) = 960 *mg*/*cm*^3^, degree of anisotropy (DA) = 0.17) and a subject of a 93 yrs (Right; Tb.BMD = 64 *mg*/*cm*^3^, Ct.Th = 0.32 *mm*, Ct.BMD = 685 *mg*/*cm*^3^, DA = 0.20). From top to bottom: A volume rendering with colors corresponding to bone mineral density values, the cortical thickness and cortical bone mineral density maps, and a reslice visualization of the segmented trabecular structures used for calculating the DA.

For a global analysis the femoral head micro-CT scan was cropped to keep only the superior medial hemisphere of the femoral head. This region of the femoral head is most consistently available in the dataset and allowed 22 subjects to be included in this analysis (Table 2). To define the interior trabecular region, a mask was drawn of the entire bone region which was eroded by 7 voxels, corresponding to 1.03 *mm*, to remove the cortex from the analysis. In case of the presence of air pockets or other artefacts, these were manually removed from the mask. Otsu’s method was again applied to the region defined by the mask to classify all voxels as either bone or marrow (Fig. 2). The DA was then be computed within this region while ignoring tissue outside of the mask.

The Tb.BMD was also calculated for this masked region using the conversion equation derived using the calibration phantom. Also the standard deviation was calculated which provides a measure of the spread or uniformity of the bone within the region.

### Cortical thickness and density

The cortex was analyzed using the Stradwin software tool (http://mi.eng.cam.ac.uk/~rwp/stradwin/) version 5.3a. This software is capable of measuring the Ct.Th on the bone surface at a subvoxel accuracy, as well as the Ct.BMD^20^. It is based on the fitting of a model of the cortex to a CT value profile taken perpendicular to the bone surface. In order to generate a map of the cortical measurements over the femoral head bone surface, a surface mesh was first constructed of the outer bone surface. Measurements were subsequently taken at every vertex of this surface mesh.

As the irregular structure of the fovea capitis femoris might negatively affect the cortical measurements, the vertices at this location were manually removed from the mesh. In this study we measured the mean cortical thickness and cortical density of every subject, as well as the standard deviation to again provide a measure of its spread.

### Statistical Analyses

**Figure 3:**
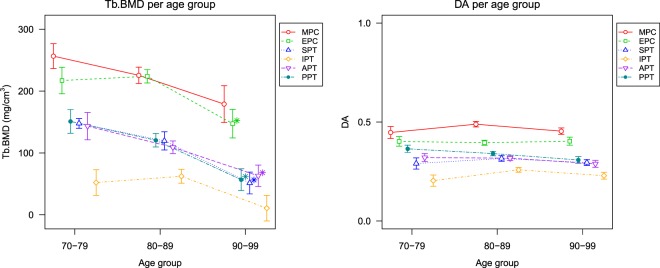
The average trabecular bone mineral density (Tb.BMD) and degree of anisotropy (DA) values at the metaphysial principal compressive (MPC), epiphysial principal compressive (EPC), superior principal tensile (SPT), inferior principal tensile (IPT), anterior principal tensile (APT) and posterior principal tensile (PPT) region as illustrated in Fig. 1. The error bars indicate the standard error of the mean. Note that for clarity the points for each region are shifted with respect to the age group markers. *Significantly different with respect to previous age group (*p* < 0.05) assessed by ANOVA, adjusted for weight and height. P-values were adjusted using Hommel’s correction to correct for multiple comparisons.

All statistical analyses were performed using R (R Foundation for Statistical Computing, Vienna, Austria). Baseline significances between consecutive age groups were assessed by two-sample T-tests. Statistical significance of the difference between age groups of the various parameters was assessed using ANOVA with type III (for unbalanced designs), adjusted for weight and height. Weight and height values are included as covariates since the load on the bone and the shape may both affect the rate and extent of the functional adaptation. To verify the normality assumption the Shapiro-Wilk test was used. When comparing parameters at the six locations (Fig. 3), the p-values were adjusted using Hommel’s correction for multiple-comparisons. Statistical significance for all tests was set at *p* < 0.05.

## Results

The results of the localized analysis of the Tb.BMD and DA are presented in Fig. 3. Between the 9th and 10th decade a significant Tb.BMD decrease is seen for the EPC, SPT, APT and PPT regions of 34.2%, 57.0%, 42.2% and 52.8% respectively. Although the decrease in the MPC and IPT regions do not reach significance after controlling for weight and height and correcting for multiple comparisons (*p* = 0.140 and *p* = 0.065 respectively), it is of the same order as the other decreases. The error bars indicate the standard error of the mean. They therefore reflect the variation of the measurements with respect to the sample size. There was no significant difference in Tb.BMD between the first two age groups. For the DA no significant change was seen between any of the groups.

**Figure 4:**
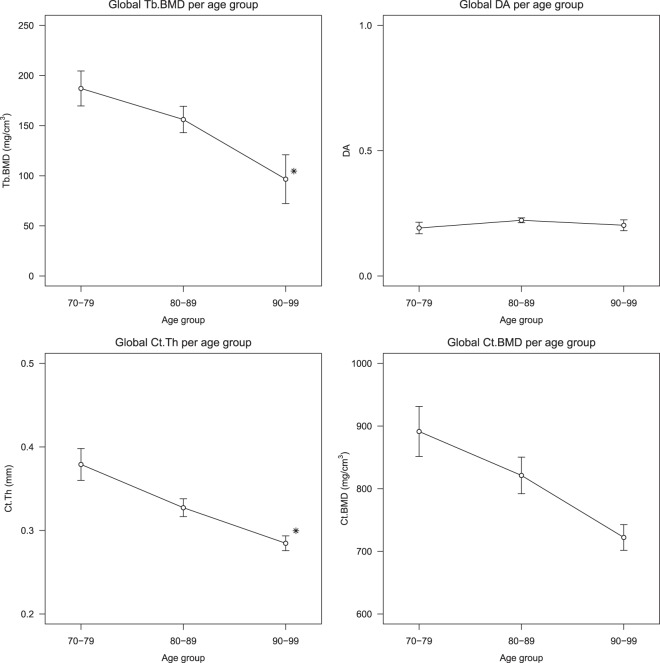
The average global trabecular bone mineral density (Tb.BMD), degree of anisotropy (DA), cortical thickness (Ct.Th) and cortical bone mineral density (Ct.BMD) values for each age group. The error bar indicates the standard error of the mean. *Significantly different with respect to previous age group (*p* < 0.05) assessed by ANOVA, adjusted for weight and height.

The global analysis indicates a significant decrease in Tb.BMD and Ct.Th (while controlling for weight and height) from the 9th to 10th decade of life (Fig. 4). For the Tb.BMD we see a decrease of 38.1% and for the cortex a thickness decrease of 13.0%. The Ct.BMD shows a decrease of 12.1%, but this change is not statistically significant (*p* = 0.056) when controlling for weight and height differences. We found no significant change in the standard deviation of the Ct.Th, Ct.BMD and Tb.BMD measurements. Thus, no change in the spread of the cortical or trabecular bone is seen, which suggests a uniform decrease throughout the femoral head.

## Discussion

When examining the global trabecular bone changes, we found a significant decrease in Tb.BMD of 38.1% from the 9th to the 10th decade of life. Previous studies report similar changes when measuring the BV/TV which, comparably to Tb.BMD, expresses the bone mineral content in the examined region or sample. Cui *et al*.^12^ showed that the BV/TV in the femoral head decreased by 22.6% from an age group ranging between 60 and 79 years to an elderly group between the age of 80 and 90 years, although this change was not statistically significant. Also Vale *et al*.^21^ report an age related reduction in BV/TV when examining the age related changes of the trabecular bone microstructure at femoral head epiphysis.

Other studies show similar changes in the femoral neck area. Chen *et al*.^14^ report a decrease of 13.2% in BV/TV at the femoral neck from subjects aged between 72 and 82 years to a group aged between 87 and 98 years. Lochmüller *et al*.^13^ showed a significant change of the femoral neck BV/TV in the female cohort with a 13.4% decrease per decade relative to a subject at an age of 60 years.

Chen *et al*.^14^ showed that the femoral neck cortical porosity increased significantly by 42.2%. This corresponds to the decrease in Ct.BMD seen in our analysis of the femoral head, since pores in the cortex are reflected by a decrease in the cortical density when measured by Stradwin^22^.

In a study by Carballido-Gamio *et al*.^23^ a thinning of the superior cortex was observed, but was less pronounced after middle-age. The authors state that the changes at this extremely thin cortical structure might be too small to be detected by clinical CT. We now show a significant decrease in cortical thickness of 13.0% per decade after the 9th decade measured from high resolution micro-CT scans, which, together with the decrease in Ct.BMD, suggests a continuing cortical bone degradation during old age.

In the work by Li *et al*.^24^ no correlation was found between the DA and age at the load bearing region of femoral heads from female osteoarthritic patients. However, this was assessed within an age range of 37 to 95 years. Lochmüller *et al*.^13^ showed that the femoral neck DA increased by 3.3% per decade in women between the age of 52 and 99 years, although this change was not statistically significant. Looking specifically at old age, Chen *et al*.^14^ report a femoral neck DA increase of 4.5% when comparing a group aged 72 to 82 years, to a group ranging in age between 87 and 98 years. However, these changes were also not statistically significant.

Cui *et al*.^12^ showed that the DA in the femoral head remained steady until the 7th decade after which it increased noticeably. This was said to indicate that the trabeculae align more strongly in the primary direction following by bone loss. However, the change in DA was only shown to be significant between the young group aged 40 to 59 years and the elderly group aged between 80 and 90 years, while the change between the old group aged 60 to 79 years and the elderly group was not statistically significant. Our results show no change in the DA at all the examined regions as well as globally within the femoral head. Thus, with also our results not indicating any reorientation of the trabecualar structures towards the principal stress trajectory, evidence of functional adaptation throughout old age remains weak.

Another way to investigate the presence functional adaptation is to examine the bone mineral density decreases at both loaded and unloaded regions. In a study by Djuric *et al*.^25^ several regions in the proximal femur were shown to change the structure differently throughout aging, but this study did not differentiate between loaded and unloaded regions. Crane *et al*.^19^ analyzed the subchondral and medial principal compressive regions using histology whereby BV/TV was shown to decrease in all regions. Our results show that changes in high stress and low stress regions within the femoral head show similar trends of bone decrease (Fig. 3). This again suggests a limited role of functional adaptation in trabecular bone at old age.

Riggs *et al*.^1^ showed that the decreases in cortical vBMD after menopause at the non-weightbearing distal radius bone was identical to those at the weight bearing distal tibia bone. The authors suggest that this indicates that the effect of estrogen deficiency is dominant over the effect of mechanical loading. Other studies suggest that the menopausal bone loss occurs only in the first few years of menopause and that bone loss after menopause is most likely caused by other factors such as nutritional inadequacies or a decreased physical activity^26^.

In the work by Stauber *et al*.^27^ a curve was fitted to BV/TV data from the femoral head using locally weighted scatterplot smoothing. No apparent changes could be seen until the age of 70, long past the average age that menopause sets in, but after this the curve shows a steep decline. Thus, functional adaptation, or more accurately, the lack thereof, may still play a considerable part in bone loss at old age. A revealing study by Allison *et al*.^28^ showed localized exercise-induced cortical bone increases across the proximal femur and at the inferior femoral head. Correspondingly, a decrease in physical activity may cause a reduction in bone and an accompanying structural deterioration. A decreased physical activity may indeed be a largely overlooked cause of age related bone decrease.

Without the presence of load adaptive remodeling to maintain bone structures in loaded regions, we would expect a uniform decrease in bone mass in the femoral head. Indeed, we found no change in the spread of the global Tb.BMD measurements or cortical parameters. Tanck *et al*.^29^ examined the distribution of the trabecular bone parameters in micro-CT scans of 7 femoral head from patients with a femoral neck fracture and 7 from patients with osteoarthrosis without a fracture. Here the results are also said to suggest that bone loss in osteoporosis occurs uniformly throughout the femoral head.

Epidemiological projections show a drastic increase in the number of hip fractures worldwide^30^. However, less reported is the shift towards an older average age at the onset of the fracture. Indeed, new data suggest an enormous burden of fractures in the over 85’s. While an increase in fracture incidence is commonly suggested to be merely a consequence of our aging society, there might be a continuing degradation of the bone structures at these extremes of age, causing a further increase in hip fracture risk. Furthermore, epidemiological reports on hip fractures, such as the widely cited study by Sambrook and Cooper^31^, predominantly report on populations reaching up to an age of 85. We have shown a significant decrease in cortical and trabecular bone throughout the 9th and 10th decade of life, which calls for further study on its cause.

Some limitations of this study should be noted. Changes seen in the femoral head might not translate directly to other regions of high fracture incidence such as the femoral neck or inter trochanter. Furthermore, the femoral head specimen used in this study were acquired from patients suffering a hip fracture. Thus, it is possible that our study population has an impaired functional adaptation. Any changes seen in this study can therefore not be considered normal aging effects and should be interpreted accordingly. As no controls were available, further studies are required to determine whether the continued decrease in bone density and the absence of the effects of functional adaptation in old age are symptomatic of an increased hip fracture risk, or seen throughout the elderly population.

To conclude, in this study, conducted on femoral head specimen of hip fracture patients, we have shown that an age-related bone degradation continues throughout old age. However, no changes in the distribution of the bone was seen and decreases show similar trends in high and low stress regions. This suggests that functional adaptation may have limited influence in maintaining structural integrity throughout old age. Whether this is due to an estrogen deficiency, a decrease in physical activity or something else entirely remains to be investigated. Thus, this study emphasizes the need for a controlled study on the cause of the continued bone degradation during old age, relating the changes to physical activity and bone turnover markers known to affect bone density. This may identify the principal factors influencing the hip fracture risk in old age and identify the appropriate therapeutic targets.
